# Characterizing alterations in the gut microbiota following postpartum weight change

**DOI:** 10.1128/msystems.00808-23

**Published:** 2023-10-31

**Authors:** Bridget N. Chalifour, Diana I. Trifonova, Elizabeth A. Holzhausen, Maximilian J. Bailey, Kelsey A. Schmidt, Mahsa Babaei, Pari Mokhtari, Michael I. Goran, Tanya L. Alderete

**Affiliations:** 1 Department of Integrative Physiology, University of Colorado Boulder, Boulder, Colorado, USA; 2 Stanford University School of Medicine, Leland Stanford Junior University, Stanford, California, USA; 3 Children’s Hospital Los Angeles, University of Southern California, Los Angeles, California, USA; Northern Arizona University, Flagstaff, Arizona, USA

**Keywords:** gut microbiome, postpartum, Latinas, obesity, network analysis

## Abstract

The postpartum period represents a dynamic time for women’s health, particularly when it comes to changes in weight, which can disproportionately affect Latina women. Weight change, particularly weight gain, has been associated with pronounced changes in host gut microbiome profiles and composition. This study aimed to evaluate changes in the gut microbiome among Latina mothers who gained or lost weight in the first 6 months after delivery by characterizing changes in gut microbial co-occurrence networks, alpha- and beta-diversity measures, and relative abundances of bacterial taxa. This study characterized the gut microbiomes of 105 Latina mothers at 1 and 6 months postpartum, comparing mothers who gained weight (*n* = 65) with those who lost weight (*n* = 40) during the 6-month period following delivery. Stool samples were collected for 16S rRNA gene sequencing. Statistical analysis was conducted using microbial co-occurrence network analysis as well as paired two-sided *t*-tests. Mothers who gained weight postpartum had significant changes in their co-occurrence network density, alpha- and beta-diversity, and relative abundances of certain microbial taxa compared to those who lost weight. Microbial co-occurrence networks, representing measures of differences in community structure dynamics, of the weight gain group decreased in density by 2%, while there was no change in density in the weight loss group. Additionally, the weight gain group had lower Shannon entropy after 6 months and significant changes in the relative abundances of 17 bacterial families and genera, while there were no significant changes in any of these metrics in the weight loss group.

**IMPORTANCE:**

Previous research has reported differences in the gut microbiome associated with varying body compositions. More specifically, within populations of mothers, the focus has been on the impact of gestational weight gain. This is the first study to examine postpartum weight change and its association with changes in the gut microbiome, similarly, it is the first to use a Latina cohort to do so. The results support the idea that weight gain may be an important factor in reducing gut microbiome network connectivity, diversity, and changing abundances of specific microbial taxa, all measures thought to impact host health. These results suggest that weight gain dynamically alters mothers’ gut microbial communities in the first 6 months postpartum, with comparatively little change in mothers who lost weight; further research is needed to examine the health consequences of such changes.

## INTRODUCTION

The prevalence of obesity in the United States has increased dramatically, with large disparities by race and ethnicity (1, 2). For example, Latino adults have a higher prevalence of obesity compared to their non-Latino counterparts, and obesity is particularly more prevalent in Latinas, with an obesity prevalence of over 50% (2, 3). At the same time, previous studies have shown the connection between obesity and the gut microbiome, with obesity being associated with pronounced changes in host microbial ecology in humans (4–6), and changes in gut microbiome composition causing increased body weight in murine models (7–9). Pregnancy is a pivotal life event that further increases susceptibility to obesity among women who do not lose their gestational weight gain during the postpartum period, especially in Latinas who are more likely to enter pregnancy overweight or with obesity (10, 11). Although the postpartum period is an important and dynamic time in women’s health, few studies have investigated how changes in body weight following birth are associated with gut microbial diversity and composition.

The human gut microbiome plays a key role in physiological processes and host immunological response (12, 13) and has also been implicated in the pathogenesis of obesity (13, 14). For instance, studies have observed differences in the composition of the gut microbiome associated with individuals with obesity as compared to lean individuals, with phylum Firmicutes increasing and phylum Bacteroidetes decreasing with increasing adiposity (4, 15). Additionally, previous studies have shown that the composition of the gut microbiome is linked to measures of body mass index (BMI), where these associations were strongest among women (11, 16–20). Furthermore, while weight loss or weight gain has been associated with alterations in gut microbiome composition and diversity (21–23), the focus has been on gestational weight gain (24–26), with no studies currently evaluating the postpartum period. The pregnancy period has been shown to induce changes in the taxonomic composition of the gut microbiome, along with measures of alpha- (a measure of microbial taxa abundances) and beta-diversity (a measure of the similarity or dissimilarity between microbial communities) (27–30). However, little is known regarding the maternal gut microbiome during the postnatal period, which can include large weight fluctuations in a relatively short time. Using high-throughput sequencing technologies, microbial communities can now be explored using measures of diversity, community dynamics, and microbial network analyses (31), contributing to knowledge of how gut bacterial composition may be linked with obesity or weight fluctuations (23, 32).

To our knowledge, no previous studies have assessed weight change and the gut microbiome in mothers during the postpartum period. It is particularly important to assess this relationship in Latinas because they are a group with a high risk for obesity and obesity-related diseases. Therefore, this study aims to evaluate the gut microbiota among Latinas that gained or lost weight in the first 6 months after delivery. We aimed to characterize the changes in gut microbial co-occurrence networks, alpha-diversity measures, and relative abundances of bacterial taxa. Ecological theory posits that more complex and interconnected microbial communities are more stable and resilient (33), and studies have shown that denser, more connected gut microbial co-occurrence networks are associated with healthier subjects (34, 35). As such, we hypothesized that the gut microbiota among mothers who gained weight would decrease in both connectivity and alpha-diversity, as well as an increase in network connectivity and alpha-diversity among mothers who lost weight. Additionally, as individual microbial taxa can provide important insights into host health (36), we also hypothesized that specific gut microbial taxa would be associated with weight gain versus weight loss.

## RESULTS

### General characteristics

We examined the gut microbial communities of 105 Latina mothers at 1 and 6 months postpartum. Among all the subjects included, the average weight at 1 month postpartum was 73.4 ± 13.03 kg, which increased to an average of 74.66 ± 14.36 kg at 6 months postpartum (*P*-value = 0.006). Of the 105 mothers, 65 (62%) gained weight from 1 to 6 months postpartum, while 40 mothers (38%) lost weight. Among those who gained weight, the average increase in weight was 4.11 kg, while the average decrease in weight among mothers who lost weight was 3.36 kg. Antibiotic usage was low in both groups with only 10% and 7.7% having reported taking antibiotics in the postpartum period among those who lost weight and gained weight, respectively. Overall, there were no significant differences between mothers who gained weight versus mothers who lost weight in characteristics including age, socioeconomic status (SES), total energy intake, and intake of carbohydrates, protein, fat, fiber, total sugar, and added sugar at either 1 or 6 months postpartum (Table 1); however, total sugar intake decreased within each weight change group, and physical activity increased significantly in the weight loss group.

**TABLE 1:** Characteristics of mother participants from the Mother’s Milk Study^*a*^

	Weight gain (*n* = 65)		Weight loss (*n* = 40)	
	1 month [mean ± SD or *N* (%)]	6 month [mean ± SD or *N* (%)]	1 month [mean ± SD or *N* (%)]	6 month [mean ± SD or *N* (%)]
Participant characteristics				
Age (years)	28.91 ± 6.37 A	29.32 ± 6.38 B	30.27 ± 6.71 A	30.65 ± 6.62 B
SES index^*b*^	26.54 ± 11.28	26.54 ± 11.28	27.74 ± 11.87	27.74 ± 11.87
Weight (kg)	74.57 ± 13.40 A	78.68 ± 14.38 B	71.50 ± 12.33 C	68.14 ± 11.84 D
Physical activity (MET min/day)^*c*^	64.08 ± 6.05 A	65.93 ± 6.89 A	65.39 ± 5.58 A	69.75 ± 8.70 B
Antibiotics (yes, no, %yes)	5, 60, 7.7%	5, 60, 7.7%	4, 36, 10%	4, 36, 10%
Healthy weight	7 (10.8%)	2 (3.1%)	10 (25%)	13 (32.5%)
Overweight	28 (43.1%)	25 (38.5%)	15 (37.5%)	15 (37.5%)
Obese	30 (46.2%)	38 (58.5%)	15 (37.5%)	12 (30%)
Average dietary intake				
Energy intake (kcal)	1,666.00 ± 515.98	1,728.94 ± 456.56	1,678.63 ± 463.10	1,664.32 ± 488.20
Protein (g/day)	72.77 ± 22.65	79.11 ± 22.13	79.36 ± 24.85	75.78 ± 23.24
Fat (g/day)	56.06 ± 22.92	61.83 ± 20.26	55.79 ± 20.32	61.64 ± 24.01
Dietary carbohydrates (g/day)	223.84 ± 76.62	218.53 ± 70.3	222.77 ± 74.56	206.40 ± 65.34
Total sugar (g/day)	97.90 ± 48.11 A	84.85 ± 36.73 B	97.99 ± 46.78 A	82.86 ± 40.67 A,B
Added sugar (g/day)	57.57 ± 34.34	45.45 ± 27.97	56.08 ± 32.85	45.98 ± 28.28
Total fiber (g/day)	16.83 ± 6.11	17.45 ± 6.86	19.33 ± 7.07	18.09 ± 7.33

^*a*^ Summary table displays descriptive characteristics and average dietary intake of 105 Latina mothers from the Southern California Mother’s Milk Study, presented as means and standard deviations (SD) or sample size and percentage of the total. Note: Letters A–D correspond to significant differences (*P*-value < 0.05) within group (1 month versus 6 months) and between groups (weight gain versus weight loss) for the corresponding variable in each row of the table. For instance, two values with the same letter denotation of “A” within a row have no significant difference but are both significantly different from those values marked with “B”; those “B” values also have no significant difference from each other. Values A–D in one row indicate that all values were significantly different from one another. No letter denotation indicates no significant difference between groups. Results shown are based on paired, two-sided *t*-tests.

^*b*^ SES, socioeconomic status; SES index is based on the four-factor Hollingshead index.

^*c*^ MET, metabolic equivalent.

### Microbial co-occurrence network connectivity decreased with postpartum weight gain

**Fig 1:**
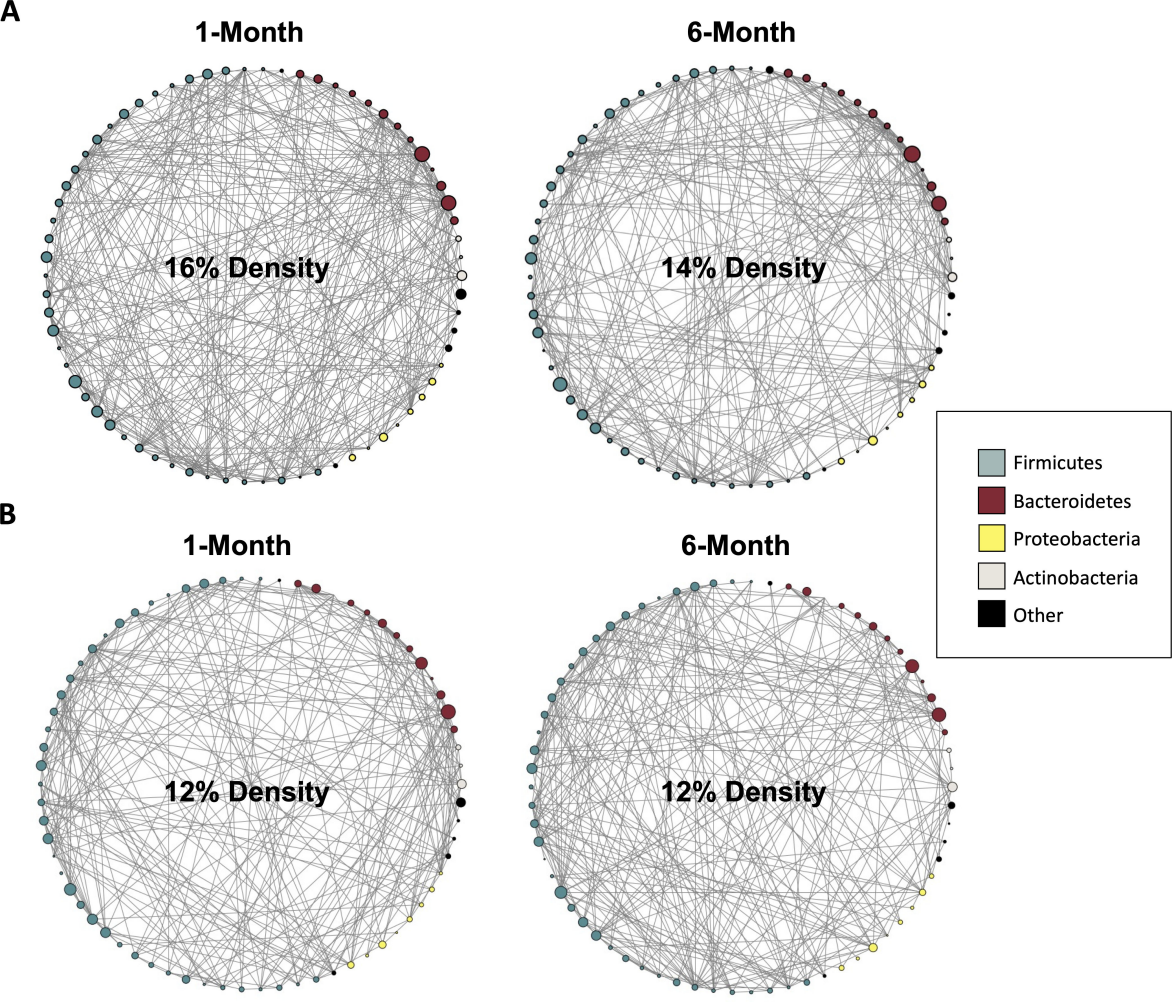
Microbial co-occurrence networks show gut microbiome community structure in mothers who gained weight (**A**) and lost weight (**B**) from 1 to 6 months postpartum. Each circle comprises nodes that represent genera within a specific phylum in the gut microbiome; node size corresponds to the logged relative abundance of the genera, and node colors represent inclusion under a certain phylum. Phyla that were not among the most abundant are reported as “Other.” Lines connecting nodes correspond to compositionally aware correlations, or edges.

Network analyses based on co-occurrence patterns were used to investigate bacterial community dynamics before and after weight change in both weight groups at the genus level (Fig. 1) and grouped by the phylum level (Fig. S1). Among those who gained weight from 1 to 6 months postpartum, gut microbial co-occurrence networks showed a decrease in network connectivity. Specifically, at 1 month postpartum, mothers who gained weight showed an average of 16% edge density in their bacterial networks, which decreased to 14% by 6 months postpartum. The number of edges between 1 and 6 months postpartum in the weight gain group also decreased from an average of 382 to 328 edges (−14.14%). Finally, the clustering coefficient, which represents the complexity of the network and the strength of the interactions among microbiota (37), remained stable at 0.41 from 1 to 6 months postpartum. In contrast, gut microbial co-occurrence networks in participants who lost weight from 1 to 6 months postpartum showed no change in network connectivity. At 1 month, mothers who lost weight showed a 12% edge density in their bacterial networks, which remained at 12% density by 6 months postpartum. The number of edges between 1 and 6 months in the weight loss group remained at 282; however, the clustering coefficient increased from 0.29 to 0.41 (41.38%).

### Gut bacterial alpha- and beta-diversity were associated with postpartum weight gain

**Fig 2:**
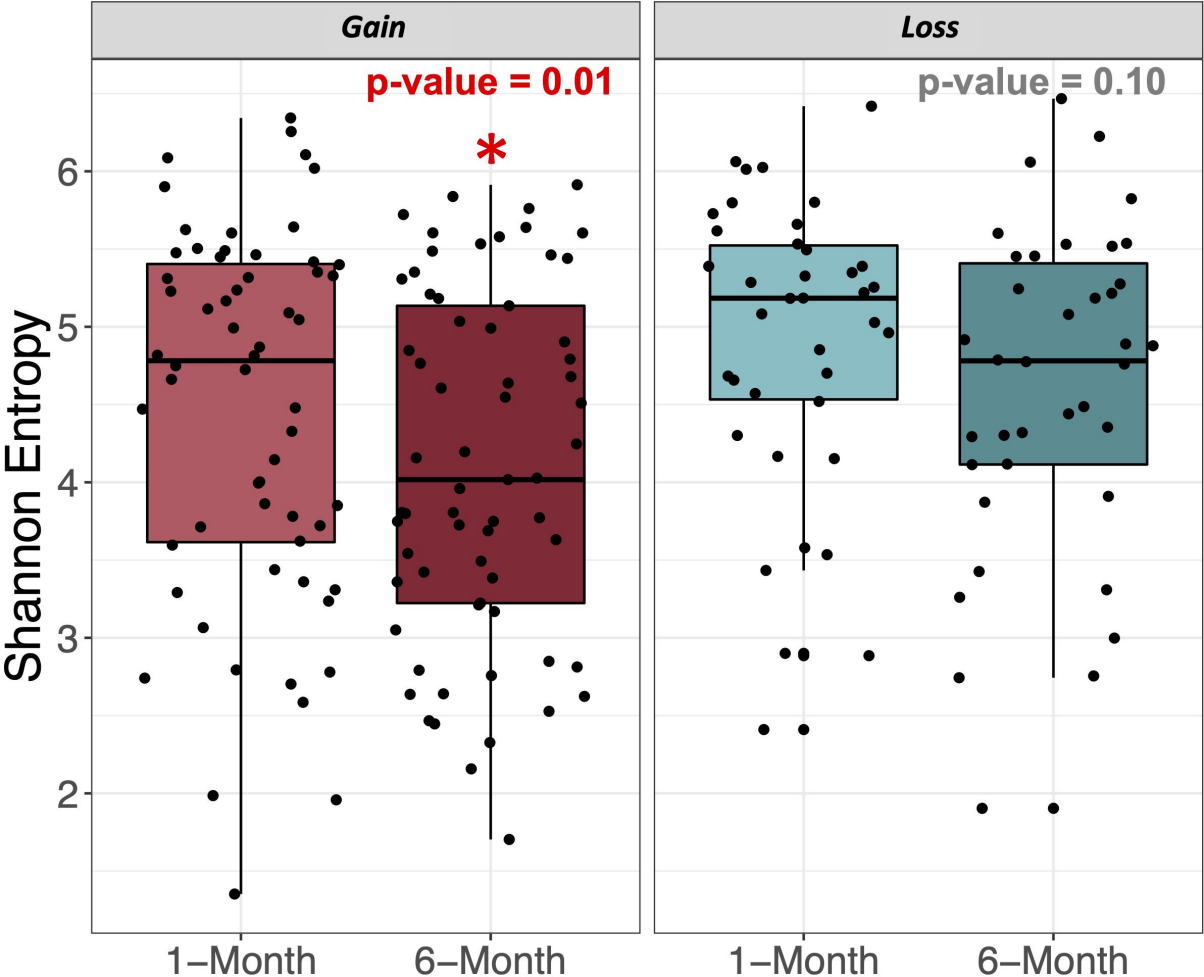
Shannon entropy significantly decreases in mothers who gained weight from 1 to 6 months postpartum. Diversity boxplots showing differences in Shannon entropy in the gut microbiomes of mothers who gained weight (“Gain”) and lost weight (“Loss”) from 1 to 6 months. Jitter shows the distribution of samples. (*) denotes significant differences between timepoints of *P*-value < 0.05.

There were significant differences in measures of alpha-diversity by weight change status (Fig. 2; Fig. S2). Alpha-diversity tended to decrease in mothers who gained weight from 1 to 6 months postpartum, and Shannon entropy significantly decreased from 4.46 ± 1.2 to 4.11 ± 1.12 (*P*-value = 0.01). Bacterial richness also tended to decrease in mothers who gained weight (*P*-value = 0.1), as did Faith’s phylogenetic diversity (*P*-value = 0.06).

Mothers who lost weight had no significant changes in measure of microbial alpha-diversity (all *P*-values ≥ 0.1). Given that there were differences in the extent of weight loss in the weight loss group, we additionally ran sensitivity analyses to determine if stratifying the weight loss group by those returning to within 5 kg (*n* = 20) or outside 5 kg (*n* = 20) of pre-pregnancy weight after 6 months postpartum led to any differences in diversity. After running these additional analyses, no differences were uncovered when stratified by the extent of weight loss; there was still no association with alpha-diversity measures in the weight loss group.

**Fig 3:**
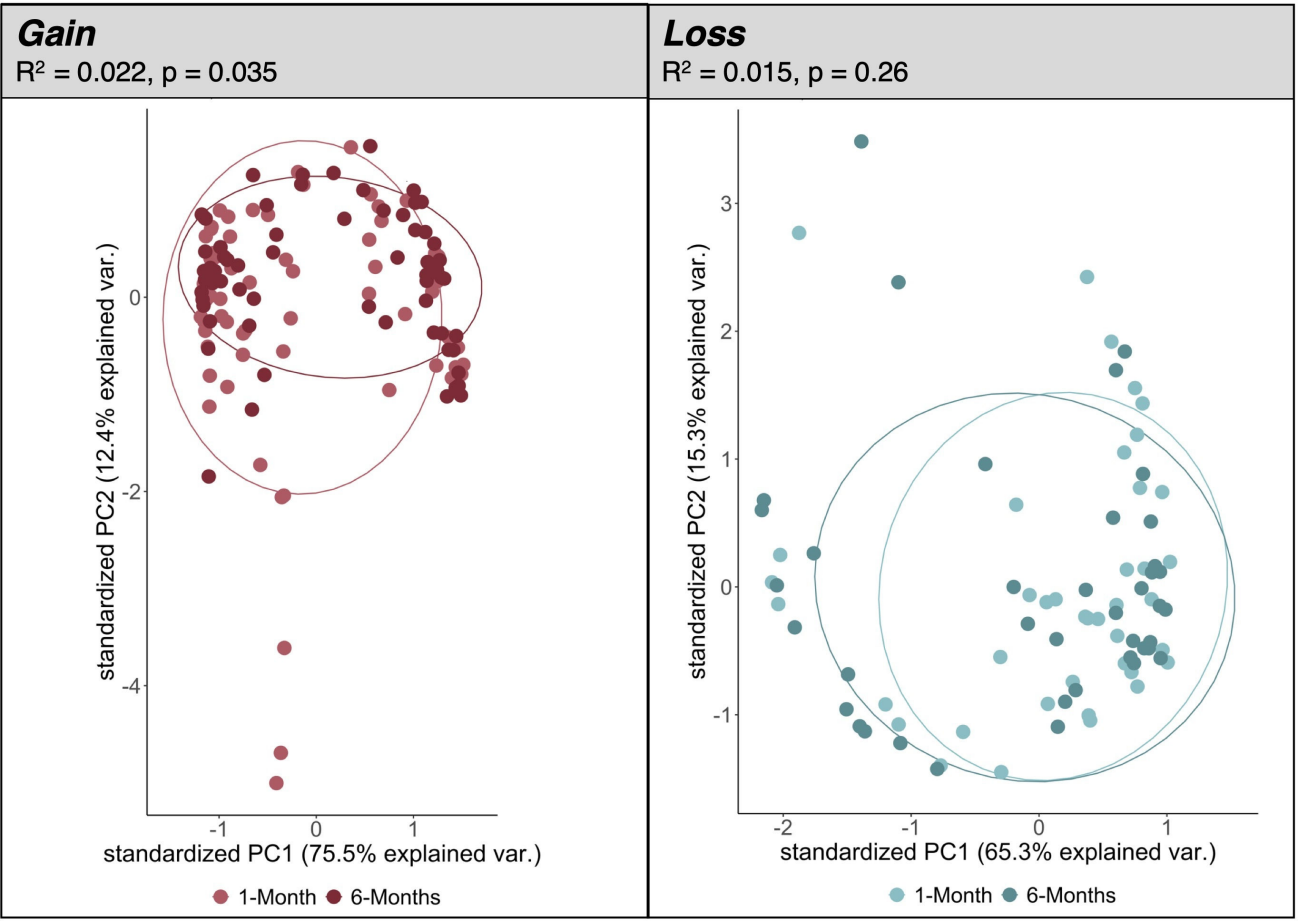
Ordination plot of principal components (PC) 1 and 2 for each weight change group. Beta-diversity is displayed by plotting samples from the weight gain group (left) and the weight loss group (right) on PC plot. Points are colored by timepoint (1 or 6 months) within each weight change group. *R^2^* and *P*-values were calculated using PERMANOVA testing. Specifically, PERMANOVA tests were performed on Bray-Curtis dissimilarity indices between timepoints within each weight change group.

Similarly, there were significant differences in measures of beta-diversity in the weight gain group only (Fig. 3). Beta-diversity did not significantly differ between 1 and 6 months in the weight loss group (*R*^2^ = 0.015, *P* = 0.26) but did change significantly between the 1- and 6- month timepoints in the group that gained weight (*R^2^* = 0.022, *P* = 0.035), with increased average beta-diversity at 6 months (0.64) compared to 1 month (0.57). We ran the same sensitivity analyses to determine if stratifying the weight loss group by those returning to within 5 kg or outside 5 kg of pre-pregnancy weight after 6 months postpartum led to any differences in beta-diversity. No differences in beta-diversity were uncovered in the weight loss group when stratifying by the extent of weight loss.

### Microbial taxa were associated with postpartum weight gain

**Fig 4:**
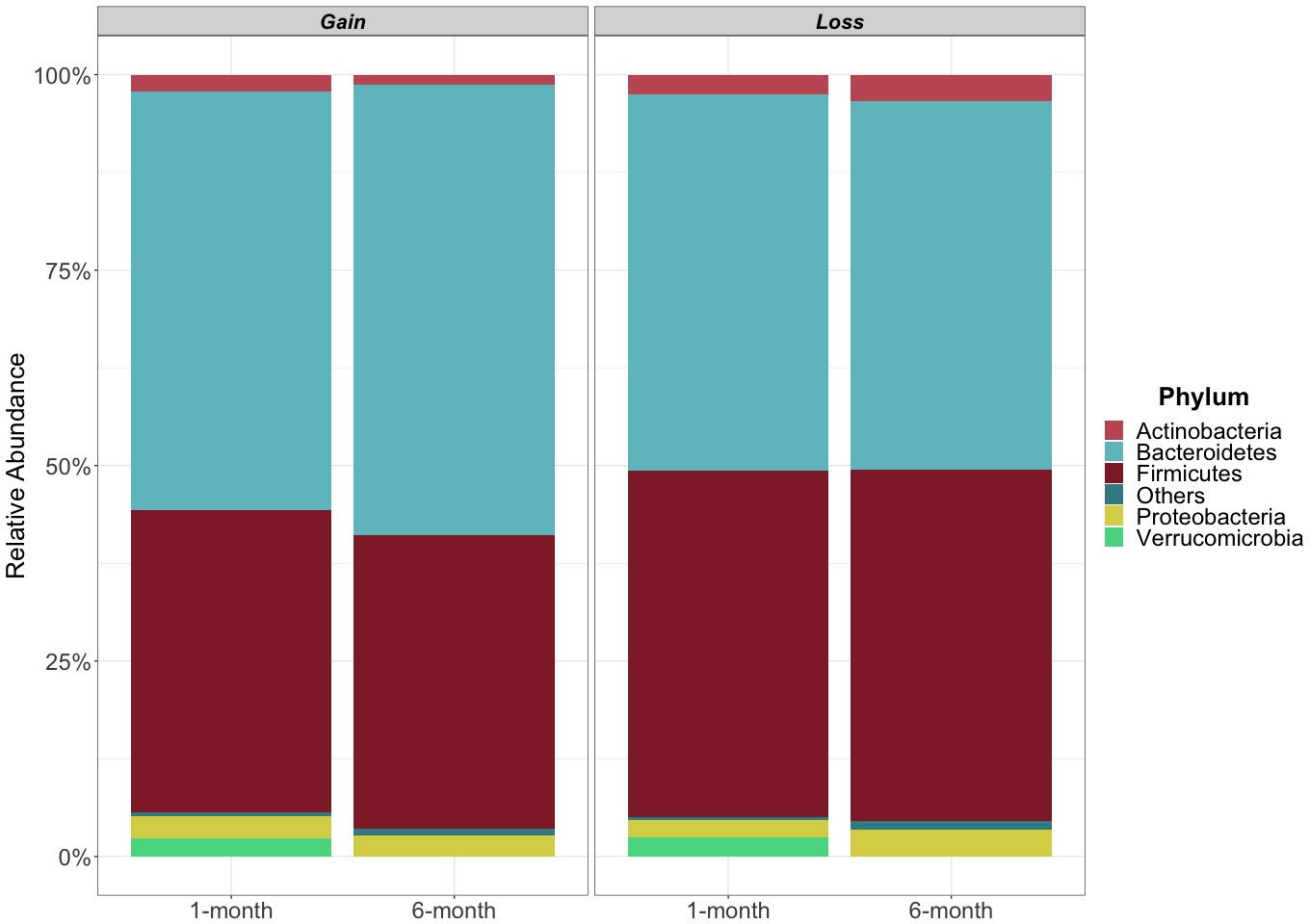
Stacked bar graphs display the relative abundance of the most abundant taxa at the phylum level in mothers who gained weight (Gain) or lost weight (Loss) at 1 and 6 months postpartum. Phyla that were not among the most abundant are reported as “Others.”

Overall, 8 unique phyla, 16 unique classes, 17 unique orders, 34 unique families, and 70 unique genera of bacteria were observed in the postpartum gut microbiota. The gut microbiota was first examined by visualizing the most abundant phyla (Fig. 4). Across the weight gain and weight loss groups at 1 month postpartum, the five most abundant phyla were Bacteroidetes, Firmicutes, Proteobacteria, Verrucomicrobia, and Actinobacteria. At 6 months postpartum, Verrucomicrobia abundance decreased in both weight change groups, leaving Bacteroidetes, Firmicutes, Proteobacteria, and Actinobacteria as the most abundant phyla.

**Fig 5:**
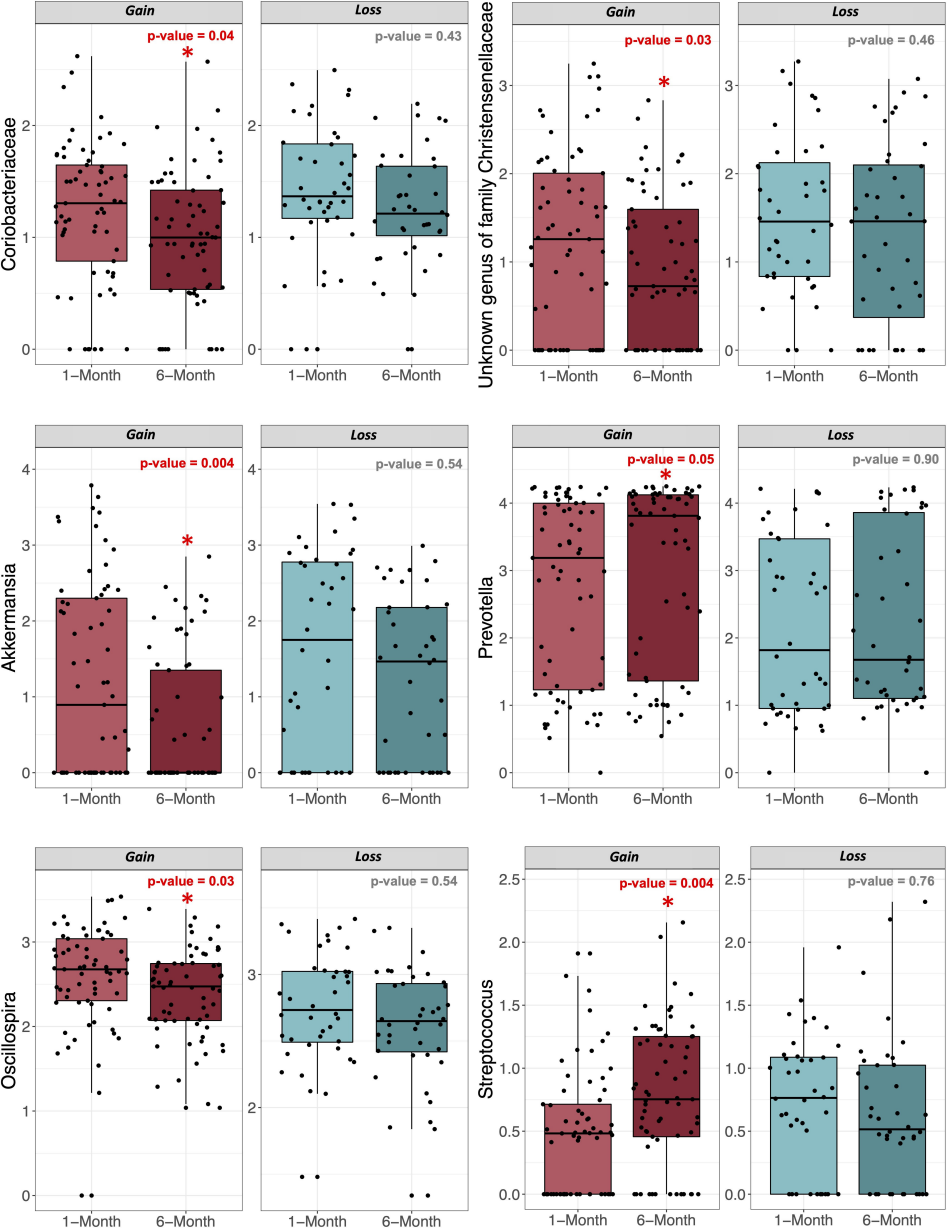
Changes in relative abundance of six bacterial families and genera grouped by weight category and timepoint. Jitter shows the distribution of samples. (*) denotes significant differences between timepoints of *P*-value < 0.05.

The change in the relative abundance of gut microbial taxa was significantly associated with weight gain from 1 to 6 months postpartum (for select taxa, Fig. 5; for all lowest-level significant taxa, Fig. S3). At the phylum level, these included Lentisphaerae, which significantly decreased with weight gain (FDR_BH_ = 0.04), and Verrucomicrobia, which also significantly decreased with weight gain (FDR_BH_ = 0.0005). Weight gain was also associated with increased abundances of gut bacterial genera belonging to phyla Bacteroidetes, Firmicutes, and Proteobacteria. Specifically, weight gain showed significant increases in the relative abundance of genera *Prevotella* (FDR_BH_ = 0.048), *Faecalibacterium* (FDR_BH_ = 0.048), *Streptococcus* (FDR_BH_ = 0.004), *Veillonella* (FDR_BH_ = 0.048), *Haemophilus* (FDR_BH_ = 0.0009)*,* and the family Pasteurellaceae (FDR_BH_ = 0.0002). Weight gain was inversely associated with the change in the relative abundance of genera belonging to phyla Actinobacteria, Bacteroidetes, Firmicutes, Lentisphaerae, Thermodesulfobacteriota, and Verrucomicrobia including *Oscillospira* (FDR_BH_ = 0.03), *Desulfovibrio* (FDR_BH_ = 0.03), unknown genera from families Mogibacteriaceae (FDR_BH_ = 0.048), Rikenellaceae (FDR_BH_ = 0.03), Christensenellaceae (FDR_BH_ = 0.03), and Cerasicoccaceae (FDR_BH_ = 0.03), and the families Coriobacteriaceae (FDR_BH_ = 0.04), Peptococcaceae (FDR_BH_ = 0.03), Victivallaceae (FDR_BH_ = 0.04), and Verrucomicrobiaceae (FDR_BH_ = 0.002). These significant, lower-level taxa associated with weight gain are reported in Table 2. These results were similar to those observed in sensitivity analyses that adjusted for changes in total sugar consumption and physical activity (Table S2).

**TABLE 2:** Changes in the relative abundances of gut bacterial genera and families belonging to dominant phyla were significantly associated with weight gain in mothers^*a*^

Phylum	Bacterial taxa	Percent change in relative abundance	FDR_BH_
Actinobacteria	Family—Coriobacteriaceae	19	0.036
Bacteroidetes	Family Mogibacteriaceae—unknown genus	24	0.048
	Family Rikenellaceae—unknown genus	20	0.031
	Genus*—Prevotella*	9	0.048
Firmicutes	Family Christensenellaceae—unknown genus	25	0.028
	Family—Peptococcaceae	37	0.034
	Genus*—Faecalibacterium*	8	0.048
	Genus*—Oscillospira*	8	0.032
	Genus*—Streptococcus*	65	0.0041
	Genus*—Veillonella*	51	0.048
Lentisphaerae	Family—Victivallaceae	36	0.036
Proteobacteria	Family—Pasteurellaceae	85	0.00022
	Genus*—Haemophilus*	84	0.00098
Thermodesulfobacteriota	Genus*—Desulfovibrio*	25	0.031
Verrucomicrobia	Family Cerasicoccaceae—unknown genus	47	0.029
	Genus*—Akkermansia*	50	0.0041

^*a*^ Summary table displays statistically significant (FDR_BH_ < 0.05) positive and negative changes between 1 and 6 months in gut bacterial taxa among mothers who gained weight. Taxa are grouped alphabetically based on the phylum to which they belong; within phyla, families are listed first, followed by genera, all alphabetically. Results shown are based on paired, two-sided *t*-tests.

Mothers who lost weight had no significant changes in the relative abundances of any gut bacterial taxa between 1 and 6 months postpartum. As previously described, we examined if these associations differed among mothers who did and did not return to within 5 kg of their pre-pregnancy weight by 6 months postpartum. Overall, there were no associations with the relative abundances of any gut bacterial taxa in the weight loss group regardless of the extent of weight loss.

## DISCUSSION

This study is the first to characterize the associations between the composition of the gut microbiota and weight changes postpartum and similarly the first to compare these associations in a cohort of Latinas. Our results showed that weight gain during the first 6 months postpartum was associated with alterations in gut microbial co-occurrence network metrics and measures of alpha-diversity, and the relative abundance of several gut bacteria usually associated with these results, along with other studies, suggests that weight change, especially weight gain, may impact gut bacterial profiles and dynamics. These findings have significant public health relevance since the gut microbiome has been linked with several adverse health outcomes, including obesity (38–42), which now impacts more than 650 million adults globally (43).

In the current study, we utilized co-occurrence networks to examine gut bacterial communities. Co-occurrence networks are an emerging method used to capture important patterns in relative abundances between different microbial taxa (44, 45). In mothers who lost weight, the clustering coefficient increased, indicating that microbial interactions became stronger from 1 to 6 months postpartum, while in mothers who gained weight, the clustering coefficient remained stable. Importantly, a well-connected network may represent a greater symbiotic state and an overall healthier gut microbiome, as having a larger number of taxa that co-occur may be indicative of a robust network that can respond easier and better to exogenous changes (34, 35). Additionally, weight gain resulted in several marked differences in gut bacterial dynamics as compared to weight gain. Specifically, in mothers who gained weight, microbial co-occurrence network density decreased by 2%, from 16% to 14%. These findings are consistent with another study examining obesity in African-origin women, which found that leaner women had significantly higher measures of network density and connectivity in their gut microbial communities than women with obesity (i.e., fewer edges and edge density) (34). In contrast, among mothers who lost weight, microbial co-occurrence network density remained the same from 1 to 6 months postpartum. This may be because mothers who lost weight started at an already lowered weight compared to mothers who gained weight, so changes in gut bacterial community connectedness may have been less dramatic. Additionally, mothers who lost weight may have already had a gut microbiome with more stable community dynamics, making the structure of this community more resilient to changes associated with the postpartum period. The inverse trend has been shown to take place in malnourished children, where children with borderline or chronic malnutrition have increased network density compared to healthy-weight children; this may be due to increasing functional interdependence among bacterial taxa (46, 47). Additionally, mothers who lost weight also had slightly lower network density at the baseline time point of 1 month. However, we emphasize the relative change in network density between 1 and 6 months as an unbiased measure of interactions and changes happening within each network.

Another factor shaping network density is the number of nodes present in the network, in this case, the number of bacterial taxa. As the density of a network is influenced by both the number of nodes and the number of connections among them, increasing the number of bacterial taxa present in analysis, whether through deeper sequencing or less stringent filtration methods, increases the number of possible connections and can result in a higher network density. Therefore, while the previously cited study by Dugas et al. does show that leaner women had significantly higher measures of network density compared to women with obesity, exact measures of network density are specific to the cohorts and data sets they are associated with. By examining species-level networks in the study by Dugas et al. (34), there is an inherent grouped nature of species found within the same genera, and it is reasonable to assume that there may be more correlations between species within the same genera. More associations between species of different genera may result in a higher edge density due to the presence of multiple species within one genus sharing patterns of connectivity. So, comparing network density alone between these studies may not provide a comprehensive understanding of the network structure and function. Future studies should strive to include larger and more similar sample sizes across weight change groups to improve network analysis performance (48) and should also be wary of comparing studies’ network analysis results without considering both sample size and number of nodes.

Previous studies have found that obesity and higher BMI tend to be associated with decreased gut bacterial diversity (49, 50) and a healthy gut microbiome has an overall high level of biodiversity (51). Our findings are consistent with this observation where Shannon entropy significantly decreased in women who gained weight from 1 to 6 months postpartum. This corroborates the findings of many other studies that also report significantly lower Shannon index in adults with obesity (5). Such findings may have important health implications given that measures of alpha-diversity in the gut microbiota, such as richness and diversity indices, have been associated with better cardiometabolic health and improvements in clinical characteristics after a diet-induced weight loss intervention (52, 53). Also, as the association between lowered alpha-diversity and higher BMI is thought to be most consistent among non-Latino white populations (49), this finding provides more information on Latino cohorts’ trends in alpha-diversity. Other measures of alpha-diversity, including gut richness and Faith’s phylogenetic diversity, also tended to decrease in the weight gain group; however, these did not reach statistical significance. Despite this, these observations are consistent with other studies that show the conditions of overweight/obesity in mothers during pregnancy are associated with lower alpha-diversity (54). However, not all studies point to lowered alpha-diversity with weight gain. One previous study noted that alpha-diversity measures were significantly lowered in mothers whose gut microbiomes were Bacteroidetes-dominated and were not associated with pre-pregnancy BMI or gestational weight gain (55).

Beta-diversity, as measured by Bray-Curtis dissimilarity, was significantly higher at 6 months in the weight gain group. Other studies have found similar trends in changes in beta-diversity, where beta-diversity metrics are positively associated with BMI (56) and gestational weight gain (57), and overall act as a signature to differentiate participants with obesity from participants with normal weight across many studies (19). A similar study conducted in Italian adults found significant differences in gut microbial Bray-Curtis dissimilarities between participants with overweight/obesity versus normal weight groups (58). The changes in beta-diversity observed in only the weight gain group across 1 and 6 months support our network analysis findings that overall gut microbial composition varies significantly between study cohorts pre- and post-weight gain.

In addition to examining gut microbiota networks and measures of diversity, we sought to determine if postpartum weight change was also associated with changes in the relative abundance of specific gut bacterial taxa. From this analysis, we found that two phyla were associated with weight gain (Lentisphaerae and Verrucomicrobia), along with many lower-taxonomic level microbiota. These two phyla have been linked with levels of obesity and weight change in both animal and human studies. Lower abundances of Lentisphaerae are associated with weight gain in cattle (59); however, no difference in abundance was reported in one study that compared gestational weight gain in mothers (55). Verrucomicrobia is observed to decrease in adults with obesity across the literature, supporting the trends found in the weight gain group in our study (60). More specifically, Verrucomicrobia is found in lower proportions in prediabetics, and increased proportions after dieting and gastric bypass surgery interventions (60).

While we identified overlapping bacterial families with previous weight change studies, some of the observed directions of change were inconsistent. For example, the family Coriobacteriaceae has been reported to increase with obesity (61), where we found that the relative abundance of Coriobacteriaceae significantly decreased with weight gain. Similar to our findings, another study reports that Coriobacteriaceae was associated with good metabolic health in overweight and obese populations and was significantly lowered in abundance in metabolically unhealthy individuals (62). Increased presence of Coriobacteriaceae is generally considered to be beneficial, as members contribute to important biological host functions such as glucose homeostasis and lipid metabolism (63, 64); therefore, lowered presence in mothers who gained weight may be indicative of poor metabolic functioning. In accordance with previous work, we found that the relative abundance of Christensenellaceae decreased among women who gained weight, which is consistent with the results showing that Christensenellaceae has been associated with increased leanness and lowered BMI in women (54), and adding members of Christensenellaceae to the gut microbiome of rats has been shown to be protective against weight gain and led to reduced adiposity measures (65). Additionally, the family Pasteurellaceae has been reported to increase in rats fed with a high-fat diet, compared to a normal diet (66); this family significantly increased in the weight gain group.

Overall, we observed changes in gut bacteria across several taxonomic levels among women who gained weight during the postpartum period, and these findings were consistent when adjusting for changes in physical activity and total sugar during the postpartum period. Previous studies support our findings regarding changes in gut bacterial genera in the weight gain group. For example, we found that *Akkermansia* relative abundance decreased with weight gain. One member of this genus, *Akkermansia muciniphila*, is a mucin-degrading bacterium and is reported to have an inverse association with obesity, where abundance decreases with increasing measures of obesity. These include body fat mass and fasting glucose in both mice and human studies; it is thought that higher levels of *Akkermansia* could actually reduce the risk for obesity (67, 68). However, even in specific bacterial species like *A. muciniphila,* the underlying mechanisms by which the taxa interact with obesity and obesity-related conditions like diabetes and atherosclerosis remain unclear (69). We also found that *Prevotella* significantly increased in the weight gain group; *Prevotella* is a genus that is well-documented to potentially be positively associated with higher BMI and obesity (70, 71). This relationship may be due to *Prevotella’*s efficiency in extracting energy from polysaccharide-rich food, which may lead to obesity in a high-calorie diet (70, 72). Additionally, *Prevotella* abundance has implications for pathogenicity, as members aid in the production of sialidase, an enzyme that degrades mucin, allowing pathogenic bacteria to invade the host (73). Again, it will be important for future studies to identify specific *Prevotella* species’ associations, as there is abundant genetic diversity within the genus. For instance, some *Prevotella* members are implicated with beneficial health effects, such as improving glucose metabolism, and others with negative health consequences, such as promoting obesity and inflammatory diseases (74). *Oscillospira* has been reported to be enriched in leaner subjects with lowered BMI in both child and adult studies (65, 75–78), and we observed a decrease in the relative abundance of this genus in the weight gain group. As *Oscillospira* has also been reported to decrease in cases of inflammatory illnesses, like Crohn’s disease, this genus may be important to understanding adverse health outcomes that may be linked to weight gain (79). We also found that *Streptococcus* significantly increased in the weight gain group. This finding is consistent with a previous study among Italian adults with obesity, which found increases in *Streptococcus* among those with obesity compared to those without obesity (58). Finally, we found that the abundance of *Desulfovibrio* significantly decreased in the weight gain group, which was similar to a study that observed lower levels of *Desulfovibrio* in children with overweight or obesity compared to their lean counterparts (80). However, another study found that members of *Desulfovibrio* were significantly enriched in children with obesity and non-alcoholic fatty liver disease (81). Similarly, in adults, a positive correlation was observed between BMI and *Desulfovibrio* (82). Thus, since not all studies find the same direction of association between *Desulfovibrio* abundance with body weight, additional work is needed to further investigate the role of *Desulfovibrio* in weight gain.

### Strengths and limitations

We believe this study is the first to examine differences in the gut microbiota in postpartum women after weight change. More specifically, it is also the first to directly study postpartum weight change in Latina women, a group with a high risk for obesity and obesity-related illness. It benefits from a larger sample size that persists longitudinally from 1 to 6 months postpartum. Additionally, women who gained and lost weight did not differ in any participant characteristic except for weight, physical activity, and total dietary sugar intake; however, differences in weight were expected given our study design. Also, we observed that women who gained and lost weight reported similar declines in total sugar consumption at 6 months compared to 1 month postpartum; similarly, both groups reported an increase in physical activity from 1 to 6 months postpartum. Furthermore, sensitivity analysis showed similar significant taxa results when accounting for changes in total sugar intake and physical activity. While we observed several taxa that changed with weight gain from 1 to 6 months, it is important to note that a change in the relative abundance of a single microbial taxon may or may not affect microbiome functionality, but can provide important empirical evidence that these taxa are specifically associated with certain health conditions (36). Additionally, this study benefits from incorporating measures of connectivity with information on individual taxa. Studying the interactions between multiple microbial taxa through co-occurrence network analysis as well as the relative abundance of taxa gives a more holistic view of the stability and resilience of the gut microbiome over time, which may more strongly shape overall host health (33).

We acknowledge several limitations in our study. First, we acknowledge that mothers who lost weight had a lower average weight at 1 month postpartum when compared to mothers who gained weight. Mothers who lost weight had their group’s weight decrease by 3.36 kg, while the weight gain group increased by 4.11 kg, on average. While these are very similar values, we acknowledge that the additional weight gained by the weight gain group may have contributed to greater differentiation between groups. We also relied on self-reported 24-hour diet recalls and questionnaires to capture the dietary components of the study, as is standard for many large study cohorts. As this study was performed in an exclusively Latina population, the generalizability of our results is limited. One important study limitation is that we were unable to examine the gut microbiome during the prenatal period, which may have partly contributed to the differences that we observed in gut bacterial network connectivity at 1 month postpartum. For instance, gut microbial diversity is thought to increase during the second trimester, and there are changes in the abundance of specific bacterial species throughout pregnancy, including taxa belonging to Bacteroides and *Prevotella* (30). Such changes may be influenced by a variety of factors, including genetics, lifestyle, and diet. Given this, research that includes assessment of the gut microbiome pre- and postnatally is needed to examine the potential effects of pregnancy-specific changes to gut bacterial communities on the gut microbiome in the postnatal period. Another important limitation of this study is the use of 16S rRNA gene sequencing, which only provides information regarding gut bacterial composition. For this reason, we referenced relevant literature to indicate the potential biological relevance of our findings, particularly for relative abundance shifts in genus-level taxa. Without species-level resolution, we can only speculate as to the reasons why these genera’s abundances shift with weight change. We suggest that future studies examine the function potential of the gut microbiome using metagenomic sequencing and/or metabolomic analysis to further elucidate the mechanisms underlying the associations described in this study. Finally, while this study indicates trends between measures of gut bacterial profiles, diversity, and composition, we are unable to determine if weight change altered the gut microbiota or vice versa.

### Future directions

Obesity and the associated increased risk for chronic disease disproportionately impact racial and ethnic minority populations, which makes studying minority cohorts and the gut microbiome an even more pressing issue (83). Results from this study suggest that variation in the gut microbiota may be associated with fluctuations in body weight, particularly in Latina women with postpartum weight gain. Further study is warranted to examine the mechanisms by which weight change may impact the structure and function of the gut microbiome and the impacts this may have on mothers’ health. Additionally, changes in the maternal gut microbiome during the postpartum period may also impact the developing gut microbiome in infants. For example, the maternal gut is thought to be a primary source of bacteria found in breast milk (84), which helps shape the infant gut microbiome through vertical transmission (85). This is important since the infant gut microbiome may contribute to childhood weight gain and obesity (86–88).

### Conclusions

This study demonstrates that metrics of the gut microbiome, including co-occurrence network density, alpha-diversity, and taxa abundances, can dynamically change alongside alterations in mothers’ weight during the 6 months postpartum. In particular, the most significant alterations are associated with mothers who gained weight postpartum, with no significant change observed among mothers who lost weight, implying that weight gain has a greater overall effect on the composition and dynamics of the gut microbiome.

## MATERIALS AND METHODS

### Study subjects

We longitudinally examined the gut microbiome among Latina mothers at 1 and 6 months postpartum from the ongoing Southern California’s Mother’s Milk Study, which is examining the associations between breast milk factors and infant growth and the gut microbiota in Latina mother-infant pairs (NIH R01 DK110793 and The Gerber Foundation). Gestational parents, henceforth referred to as “mothers,” were recruited between 2016 and 2017 from clinics in Southern California. At the time of analysis, 105 mothers out of 219 had complete measures of BMI and assessment of the gut microbiota at 1 and 6 months postpartum and were included in this analysis. Overall, included mothers were a subset of all mothers that had available microbiome data and had completed visits at 1 and 6 months postpartum; these differences in obesity status and diet were due to chance. Those individuals included in the current analysis were similar to those who were excluded; however, those included had slightly lower BMI, total energy intake, and other nutrients (Table S1).

The design of the Mother’s Milk Study has been described in detail in previous work (89–92). Briefly, study participants were recruited from maternity clinics affiliated with the University of Southern California in Los Angeles County and other community clinics. The eligibility criteria included: having a healthy, term, singleton birth; self-identified Hispanic aged ≥18 years old at the time of delivery; with study visits at 1 and 6 months postpartum. Participants were excluded if they were diagnosed with any medical condition possibly affecting metabolism, nutritional status, physical or mental health; taking medications that affect body weight/composition, insulin resistance, or lipid profiles; currently using tobacco or other recreational drugs; or diagnosed with fetal abnormalities. Written, informed consent was obtained from all participants, and the University of Southern California, Children’s Hospital Los Angeles, and the University of Colorado Boulder Institutional Review Boards approved the study protocol.

### Study visits

Mothers’ weight was measured to the nearest 0.1 kg (Tanita, Model BC-549), and standing height was measured to the nearest 1 mm (Seca GmBH & Co. KG, Model Seca 126) to calculate BMI (kg/m^2^) (93). Dietary information including intake of energy, protein, fat, carbohydrates, total sugars, added sugars, and total fiber was assessed using 24-h diet recalls using Nutrition Data System for Research questionnaires at 1 and 6 months postpartum; methods of conducting these recalls are described in detail in reference (89). Physical activity information was collected using 3-day physical activity recalls to examine the number of metabolic equivalent (MET) minutes performed per day. Antibiotic usage was assessed via questionnaire; an answer of yes indicated that mothers had received any antibiotics since giving birth, and an answer of no indicated that they had not received any antibiotics since giving birth. Information regarding education and occupation was collected via a questionnaire to calculate individual socioeconomic status (SES) based on a modified version of the four-factor Hollingshead Index (94).

### Gut microbiota analysis

Stool samples from mothers were collected using OmniGene GUT kits at 1- and 6-month postpartum visits. DNA was extracted from stool samples using the Qiagen MagAttract PowerSoil DNA KF kit (384), and the V4 hypervariable region of the bacterial 16S rRNA gene was amplified by PCR using the 515/806 barcoded primer pair [515F (Parada): GTGYCAGCMGCCGCGGTAA, 806R (Apprill): GGACTACNVGGGTWTCTAAT]. DNA was then standardized in accordance with the Earth Microbiome Project (95). Paired-end, 2 × 150 bp, next-generation sequencing was performed on the Illumina MiSeq platform available in the Institute for Genomic Medicine at the University of California San Diego. Negative and extraction controls in the form of blanks were included throughout the amplification and sequencing process. Demultiplexed files were processed using Qiita (96). Sequences were trimmed to a length of 150 bp, and Deblur (97) was used to remove suspected error sequences and assign amplicon sequence variants called sub-operational taxonomic units (sOTUs). Deblur tag sequences were taxonomically assigned using the GREENGENES reference database (version 13.8) and SATÉ-enabled phylogenetic placement. Within the Quantitative Insights Into Microbial Ecology 2 (QIIME2) pipeline, a feature table was generated with counts of each sOTU for each sample. Initial library size ranged from 12 to 64,069 reads in each maternal sample, with a median of 30,785 reads and an average of 30,615 reads. The distribution of library size for each maternal sample is shown in Fig. S4. For metrics related to gut bacterial community diversity, we normalized the sequencing depth across samples by rarefying the number of reads per sample to a read depth of 10,000 which resulted in nine samples being dropped. For alpha-diversity (within-sample diversity), we estimated sOTU richness, Shannon diversity, and Faith’s phylogenetic diversity based on these rarefied samples.

Prior to transforming taxa counts to relative abundance, taxa with less than or equal to 420 total counts were filtered out of the data set. The threshold of 420 counts was determined based on an average of two reads per sample (210 samples from 105 participants at 1 and 6 months). Taxa that were observed in less than 10% of samples were also removed from the data set. The relative abundances of gut microbial taxa at various taxonomic levels, ranging from phylum (*n* = 8) to genus (*n* = 70), were examined. The relative abundances of individual taxa were log-transformed to satisfy the statistical assumptions (equation 1).

General equation for log transformed relative abundance


(1) $$Log_{10}\left(\left[\frac{Raw\;count\;in\;sample\;\left(i\right)}{\#\;of\;sequences\;in\;sample\;\left(i\right)}*\;Average\;number\;of\;sequences\;per\;sample\right]+1\;\right)$$

### Statistical analysis

#### Population descriptives

Participant characteristics and descriptive statistics were examined using means and standard deviations for continuous variables and frequencies, and percentages for categorical variables and paired *t*-tests were used to determine significance within and between groups’ means.

#### Network analysis

Network analysis was conducted to examine the relationship between weight gain and weight loss with microbial community dynamics. Co-occurrence network analysis was used to investigate the connectivity between the taxa in the gut microbiomes of mothers at 1 and 6 months postpartum. For the mothers who lost weight from 1 to 6 months, a co-occurrence network was generated using FastSpar (98), the implementation of the SparCC algorithm (99) in C++ (version 11.0.3) for each timepoint. Network analysis was performed with the NetworkX package (version 2.6.3) in Python (version 3.9.7). The input for FastSpar was absolute sOTU counts after further filtering out over all samples rare taxa with less than 420 counts and sOTUs that were on average represented by less than two reads per sample, following protocol from reference (99). The output was a matrix of *P*-values, which represents the statistical significance of genera co-occurring. This *P*-value matrix was used to create network visuals and calculate statistics in Python. The co-occurrence network included each node as an individual taxon (a total of 70 nodes used) and each edge was a correlation between nodes. An edge was drawn between two nodes if the *P*-value in the correlation matrix was <0.05. Co-occurrence network analysis has been shown to become more sensitive and specific with an increasing number of samples used (100). Our sample included 65 mothers in the weight gain group and 40 mothers in the weight loss group. This difference in sample size in turn can affect the comparative interpretation of the constructed co-occurrence networks. The network constructed from a larger sample may appear denser as there are 25 more participants than the weight loss group. The inclusion of these samples increases the variance in observed counts of rare taxa, which likely increases the observed number of correlations, thus making the co-occurrence matrix less sparse. In order to account for this difference in sample size and generate more comparable network metrics, the same process was replicated in the weight gain group as was in the weight loss group. However, to account for unequal sample sizes, a subset of 40 samples from the 65 samples in the weight gain group were randomly generated 250 times without replacement to match the sample size of 40 present in the weight loss group. These randomly generated sample sets were used to permute 250 co-occurrence networks. In the case that a sample resulted in a network that was unable to be constructed due to singular values for taxa across the random sample, this sample was thrown out, a new random sample of 40 was generated, and a corresponding network was constructed. Histograms of all network analysis measures from each random sample were visualized in order to examine estimation stability. Largely, these estimates were normally distributed with no evidence of multimodality. We report the average network analysis measures (edge density, clustering coefficient, and number of edges) of these 250 networks and selected a representative network for Fig. 1 by using one of the 250 generated networks that most closely matched the observed average network statistics. Additionally, for comparative purposes, we show the results of the same analysis using the full sample size of 65 mothers in Fig. S5.

#### Diversity and gut microbial abundance analysis

To determine measures of alpha-diversity, we estimated microbial richness, Shannon entropy (where a higher value indicates higher diversity), and Faith’s phylogenetic diversity using QIIME2 (101, 102). Differences in alpha-diversity over the 6-month period within each weight change group were examined using a two-sided paired *t*-test. Beta-diversity metrics were used to compare differences in the community composition of two different samples. Bray-Curtis dissimilarity was used to compare the abundance of each taxon between two samples to give a metric between 0 (complete similarity) and 1 (no shared taxa between communities). Differences in beta-diversity based on our established timepoints of 1 and 6 months postpartum within each weight change group were examined using a permutational multivariate analysis of variance (PERMANOVA). A two-sided paired *t*-test was used to determine the significance in percent change of the relative abundances of bacterial taxa from 1 to 6 months postpartum in the weight gain group and the weight loss group. Given that we observed temporal differences in physical activity and total sugar intake within each group, we ran sensitivity analyses. Briefly, we used linear mixed models to examine how time (denoted as the 1- and 6-month visits) was associated with the change in relative abundance in gut bacterial taxa, after adjusting for change in total sugar intake or physical activity as a fixed effect and using participant ID as a random effect. As there was a low proportion of participants who used antibiotics at 1 month postpartum, we did not adjust for antibiotic usage. Additionally, we performed additional sensitivity analyses to determine if the degree of weight loss was associated with changes in alpha-diversity, beta-diversity, or taxa abundances. To accomplish this, we examined mothers who returned to within 5 kg of their pre-pregnancy weight and those who did not. In determining the differences in relative abundance in bacterial taxa, statistical analysis was corrected for multiple testing using the Benjamini-Hochberg procedure (FDR_BH_ < 0.05). Statistical analyses were conducted using R (version 4.1.1), and Python (version 3.9.7). Statistical significance was defined as a *P*-value < 0.05.

## Data Availability

The data that support the findings of this study are available on request from the corresponding author, T.L.A. The data are not publicly available, as the deposition of de-identified sequences into a public database was not included in the original informed consent for the Mother’s Milk Study. The STORMS checklist associated with this analysis is available at https://doi.org/10.6084/m9.figshare.21545448.v1 (103).

## SUPPLEMENTAL MATERIAL

Figure S1 msystems.00808-23-s0001.tif Network plots by phyla.

Figure S2 msystems.00808-23-s0002.tif Diversity box plots.

Figure S3 msystems.00808-23-s0003.tif All taxon box plots.

Figure S4 msystems.00808-23-s0004.tif Library size histogram.

Figure S5 msystems.00808-23-s0005.tif Network plots from all 65 weight-gaining mothers.

Table S1 msystems.00808-23-s0006.pdf Included/excluded participant characteristics.

Table S2 msystems.00808-23-s0007.pdf Taxa from sensitivity analysis.

ASM does not own the copyrights to Supplemental Material that may be linked to, or accessed through, an article. The authors have granted ASM a non-exclusive, world-wide license to publish the Supplemental Material files. Please contact the corresponding author directly for reuse.
