# Supplementary figures and images for: Characterizing alterations in the gut microbiota following postpartum weight change
Source: mSystems. 2023 Oct 31;8(6):e00808-23. doi: 10.1128/msystems.00808-23 (PMC10734492; doi:10.1128/msystems.00808-23)

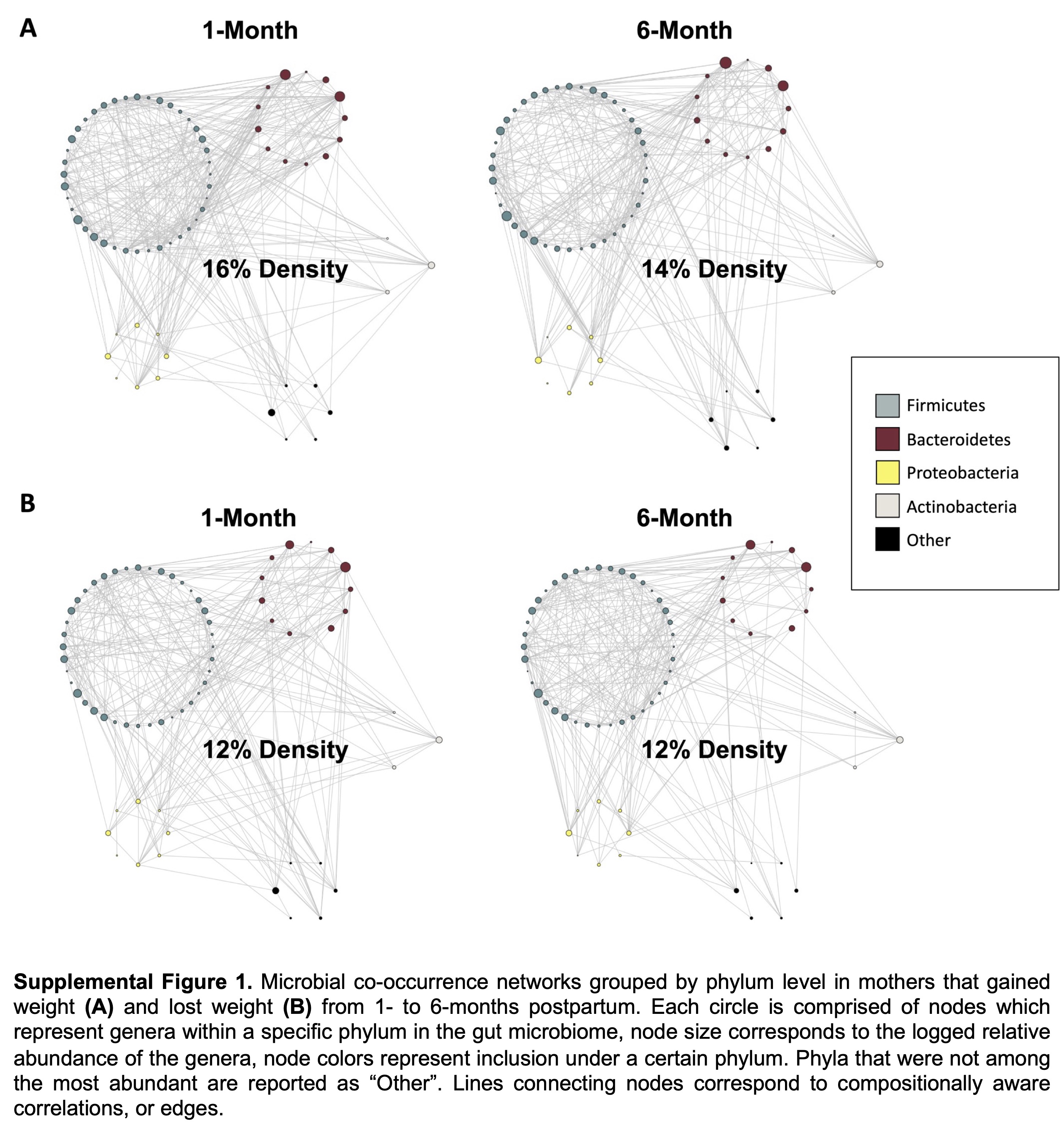

Supplement: Figure S1 — Network plots by phyla. [file msystems.00808-23-s0001.tif]

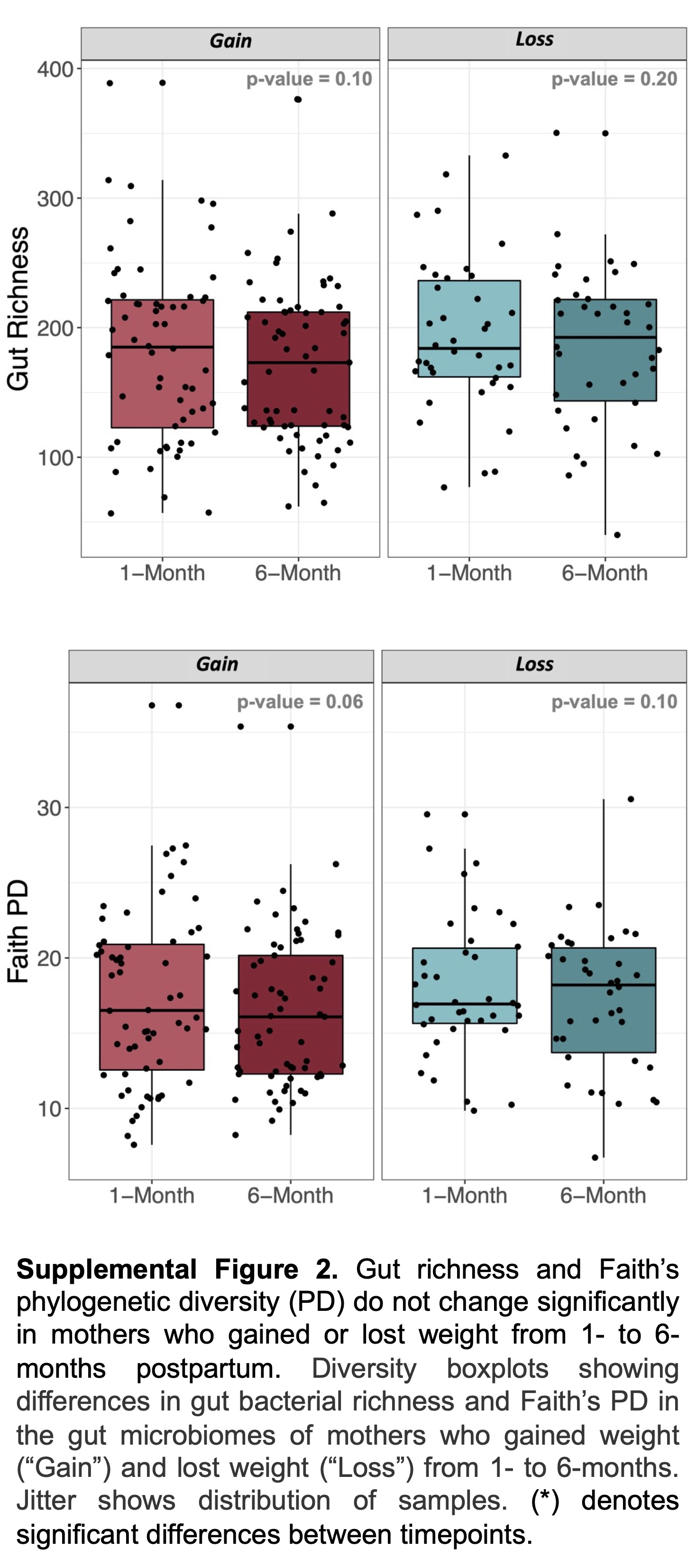

Supplement: Figure S2 — Diversity box plots. [file msystems.00808-23-s0002.tif]

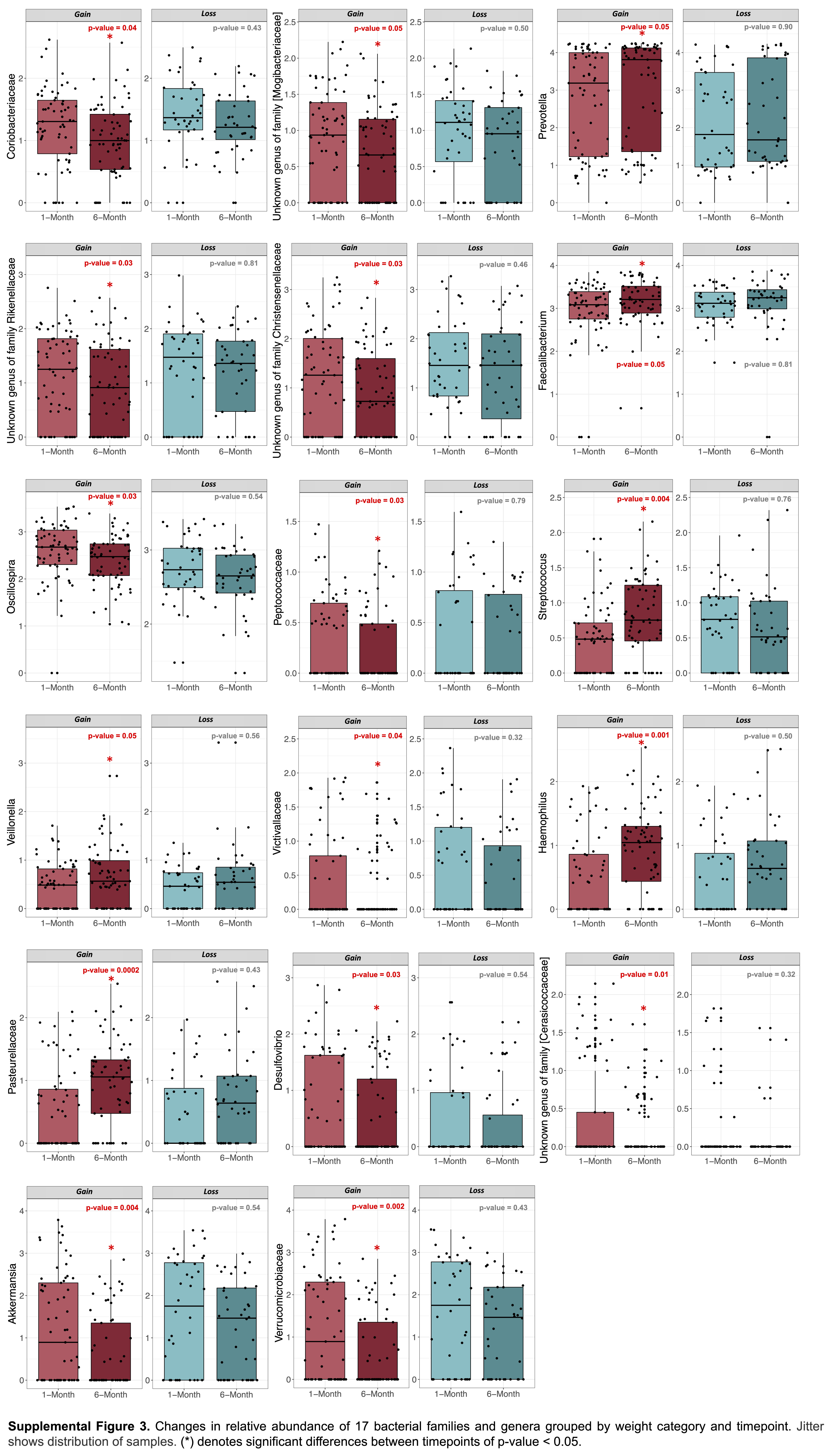

Supplement: Figure S3 — All taxon box plots. [file msystems.00808-23-s0003.tif]

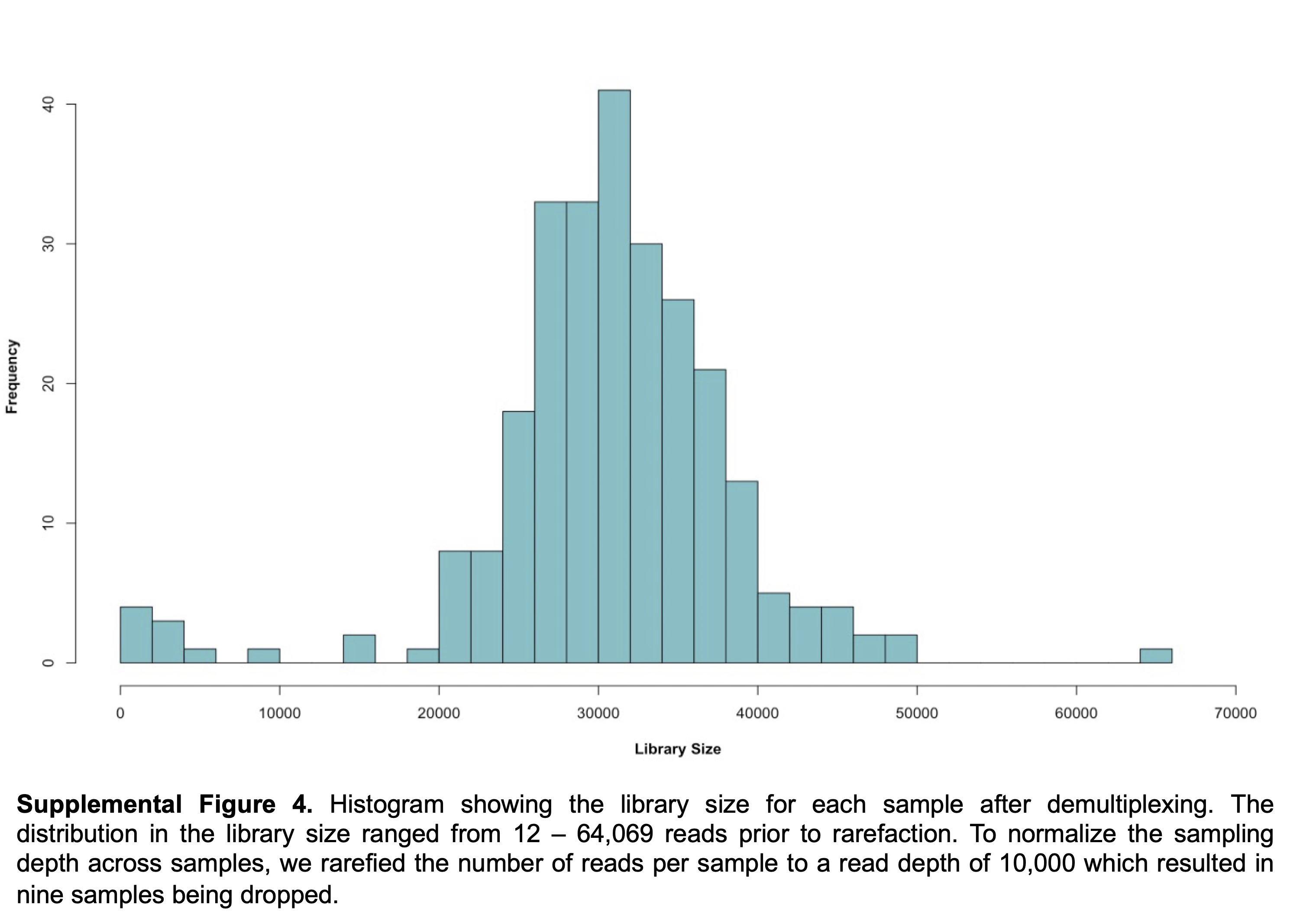

Supplement: Figure S4 — Library size histogram. [file msystems.00808-23-s0004.tif]

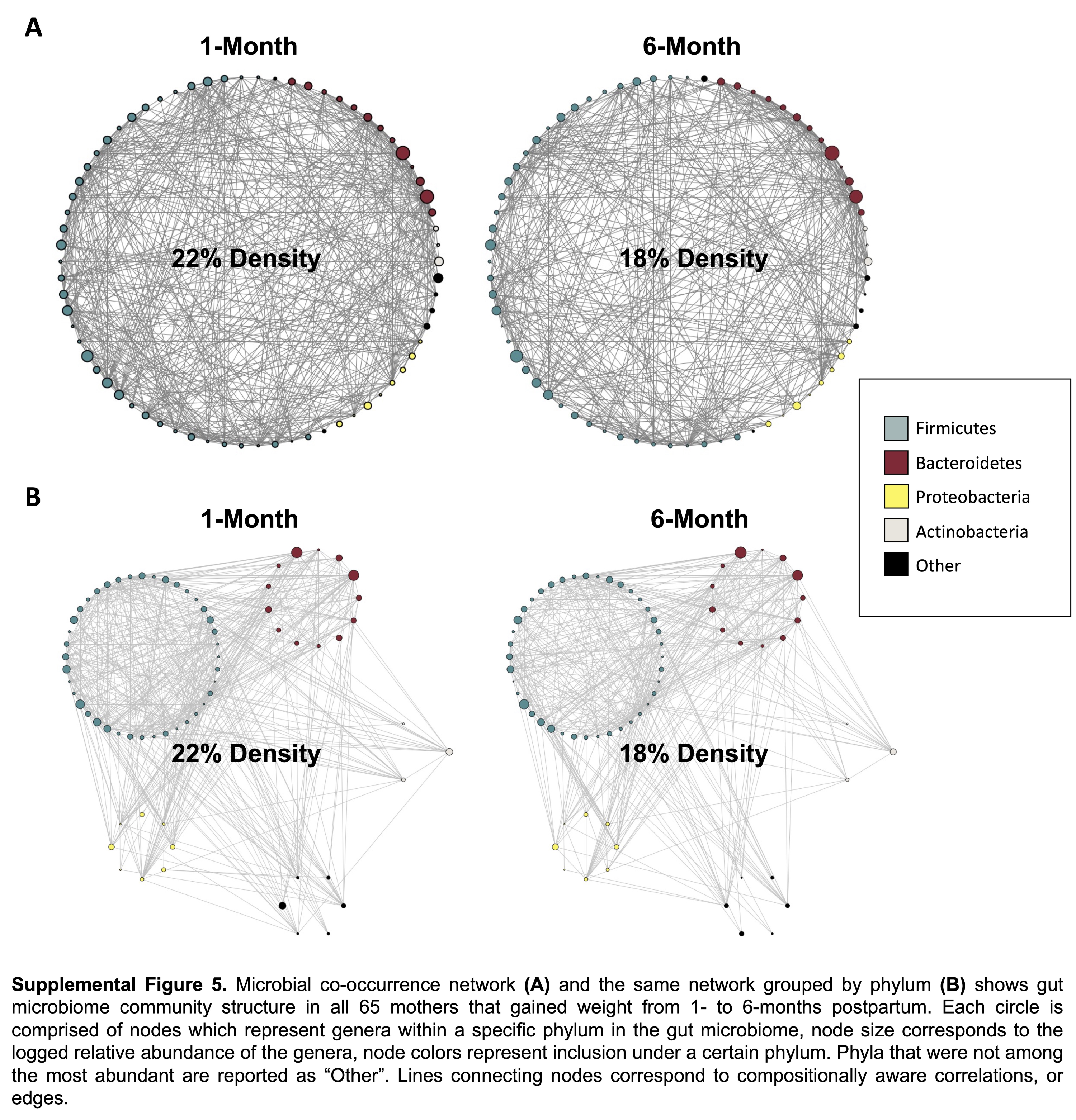

Supplement: Figure S5 — Network plots from all 65 weight-gaining mothers. [file msystems.00808-23-s0005.tif]
