# Supplementary material for: Characterizing alterations in the gut microbiota following postpartum weight change
Source: mSystems. 2023 Oct 31;8(6):e00808-23. doi: 10.1128/msystems.00808-23 (PMC10734492; doi:10.1128/msystems.00808-23)
Supplement: Table S1 — Included/excluded participant characteristics. [file msystems.00808-23-s0006.pdf]

Supplemental Table 1. Characteristics of Mothers Included and Excluded in the Analysis.

	Included in Analysis<br>Mean ± SD or N, %<br>N = 105	Excluded from Analysis<br>Mean ± SD or N, %<br>N = 114
Participant Characteristics		
Age (years)	29.43 ± 6.5[A]	28.46 ± 5.67[A]
SES Index*	27.0 ± 11.46[A]	25.99 ± 12.92[A]
Weight (kg)	28.70 ± 6.0[A]	28.4 ± 5.9[A]
Physical Activity (MET mins/day)†	64.58 ± 5.88[A]	64.63 ± 5.83[A]
Antibiotics (Yes, No, %Yes)	9, 96, 8.6%	14, 87, 12.2%
Healthy Weight	17 (16.2%)	18 (15.8%)
Overweight	43 (41%)	29 (25.4%)
Obese	45 (42.9%)	57 (50%)
Average Dietary Intake		
Energy Intake (kcal)	1670.81 ± 494.27[A]	1930.56 ± 648.86[B]
Protein (g/d)	75.28 ± 23.61[A]	82.18 ± 26.39[B]
Fat (g/d)	55.96 ± 21.87[A]	68 ± 29.36[B]
Dietary Carbohydrates (g/d)	223.43 ± 75.48[A]	254.27 ± 96.62[B]
Total Sugar (g/d)	97.94 ± 47.38[A]	108.59 ± 55.41[A]
Added Sugar (g/d)	52.96 ± 32.46[A]	65.46 ± 47.17[B]
Total Fiber (g/d)	17.78 ± 6.57[A]	19.36 ± 7.65[A]

Supplemental Table 1. Mothers who were included in the present analysis differed significantly at baseline from those who were excluded due to loss to follow-up and missing microbiome data only in some dietary intake measures. Summary table displays descriptive characteristics and average dietary intake of 219 Latina mothers from the Southern California Mother's Milk Study stratified by inclusion into the current analysis, presented as means and standard deviations (SD) or sample size and percentage of total. Letters correspond to significant differences (p-value < 0.05) between groups (included vs. excluded) for the corresponding variable in each row of the table. The same letter denotation indicates no significant difference between groups. *SES is abbreviation for socioeconomic status; SES Index is based on the four-factor Hollingshead Index. † MET is an abbreviation for metabolic equivalent. Mothers excluded from analysis had 13 missing values for antibiotics usage, and 10 missing values for BMI.
