# Supplementary material for: Characterizing alterations in the gut microbiota following postpartum weight change
Source: mSystems. 2023 Oct 31;8(6):e00808-23. doi: 10.1128/msystems.00808-23 (PMC10734492; doi:10.1128/msystems.00808-23)
Supplement: Table S2 — Taxa from sensitivity analysis. [file msystems.00808-23-s0007.pdf]

Supplemental Table 2. Similar Gut Bacterial Genera and Families were Significantly Associated with Weight Gain in Mothers After Adjusting for Total Sugar Intake and Physical Activity.

Phylum	Bacterial Taxa	FDR_{BH} – adj. for physical activity	FDR_{BH} – adj. for total sugar
Bacteroidetes	Family Mogibacteriaceae – Unknown genus	0.040	NA
	Family Rikenellaceae - Unknown genus	0.030	0.034
	Genus - *Prevotella*	NA	0.034
Firmicutes	Family Christensenellaceae - Unknown genus	0.031	0.031
	Genus - *Faecalibacterium*	NA	0.049
	Genus - *Oscillospira*	0.030	0.049
	Genus – *Streptococcus*	0.0052	0.0086
Lentisphaerae	Family – Victivallaceae	0.033	0.029
Proteobacteria	Family - Pasteurellaceae	0.00048	0.00070
	Genus – *Haemophilus*	0.0024	0.0026
Thermodesulfobacteriota	Genus – *Desulfovibrio*	0.030	0.011
Verrucomicrobia	Family Cerasicoccaceae – Unknown genus	0.027	0.016
	Genus – *Akkermansia*	0.0043	0.0086

Supplemental Table 2. Summary table displays statistically significant ($FDR_{BH} < 0.05$) positive and negative changes between 1- to 6-months in gut bacterial taxa among mothers who gained weight. Taxa are grouped alphabetically based on the phylum in which they belong, within phyla, families are listed first, followed by genera, all alphabetically. Results shown are based on linear mixed models adjusting for total sugar intake with a random effect of participant ID.
